# Maternal and perinatal outcomes of Somali migrant women in comparison to host populations in the Global North: a systematic review and meta-analysis

**DOI:** 10.1186/s40748-025-00210-1

**Published:** 2025-06-03

**Authors:** Muna Said, Itohan Osayande, Okikiolu Badejo, Aduragbemi Banke-Thomas

**Affiliations:** 1 School of Human Sciences, University of Greenwich, London, UK; 2 Department of Public Health, Institute of Tropical Medicine, Antwerp, Belgium; 3 Faculty of Epidemiology and Population Health, London School of Hygiene and Tropical Medicine, London, UK

**Keywords:** Pregnancy outcomes, Perinatal, Maternal, Somali, Migration, Meta-analysis

## Abstract

**Background:**

The enduring conflict in Somalia has precipitated significant humanitarian crises, including severely weakened health systems and poor health indicators. The situation has led to almost two million Somalis living abroad, often as refugees or asylum seekers in more high-resource settings in the Global North. To understand outcomes of care of pregnant women and their babies in host countries, this systematic review and meta-analysis aims to synthesise existing evidence on adverse maternal and perinatal outcomes among Somali migrant women compared to host populations.

**Methods:**

We conducted a comprehensive search across multiple electronic databases, including PubMed, Scopus, CINAHL Plus, and the Directory of Open Access Journals, using tailored keyword combinations. No language or date restrictions were applied, and the search concluded on June 30, 2024. Following data extraction and quality assurance using the STROBE Checklist, we conducted a meta-analysis for outcomes with sufficient data, using a random-effects model to account for heterogeneity across populations. Subgroup analyses were conducted by host country, with heterogeneity assessed using I^2^ and τ^2^ statistics. Potential publication bias was evaluated through Egger’s test and funnel plots. The results provide pooled estimates of maternal and perinatal outcomes.

**Results:**

﻿Across all databases, 116 articles were retrieved, with 17 meeting the eligibility criteria. From these articles, pregnancy-related data from 1978 to 2018 on 55,119 Somali migrant women and 5,190,459 women from the host population was extracted. Somali migrant women, compared to host populations, had significantly increased odds of emergency caesarean section (CS) (pooled OR 2.54, 95%CI: 2.22–2.86), non-progressing/induced labour (pooled OR 1.25, 95%CI: 1.19–1.31). Their babies had higher odds of small for gestational age (SGA) (pooled OR 2.03, 95%CI: 1.89–2.17), neonatal morbidity (pooled OR 1.51, 95%CI: 1.40–1.61), and neonatal mortality (pooled OR 1.39, 95%CI: 1.25–1.54). Conversely, Somali migrant women had lower odds of assisted instrumental delivery (OR 0.72, 95%CI: 0.66–0.78), post-partum depression (OR 0.27, 95% CI: 0.12–0.63), preterm birth (OR 0.92, 95%CI: 0.88–0.96), and low birth weight (OR 0.87, 95% CI: 0.80–0.94) compared to host populations.

**Conclusion:**

Significant disparities in maternal and perinatal outcomes between Somali migrant women and host populations exist. Though more research is needed, available evidence points to the need for more culturally aware obstetric services that address the specific needs of Somali migrant women.

**Supplementary Information:**

The online version contains supplementary material available at 10.1186/s40748-025-00210-1.

## Introduction

Somalia has experienced almost three decades of war and political instability. This has led to Somalia having very weak health systems and some of the worst health indicators globally [1]. For example, maternal mortality ratio in Somalia was estimated as 621 per 100,000 livebirths in 2020 with 28 stillbirths per 1,000 livebirths reported in 2021 [2, 3]. The unrest has also resulted in Somalis suffering significant losses and enduring traumatic experiences related to torture, rape, and other forms of violence [4]. Consequently, approximately two million Somalis live abroad with many living as immigrants, refugees, and asylum seekers in countries with more developed and stable economies in the Global North mostly in the Northern hemisphere, such as the United States of America (USA), Sweden, Norway, the United Kingdom (UK), Finland, and Australia [5, 6]. For example, over 103,000 Somali refugees have resettled in the USA between 2002 and 2018. Since July 2021, member countries of the European Union (EU +) have issued approximately 23,300 decisions at first instance on Somali refugee status applications [7, 8]. As of March 2023, Somali nationals were the 14th largest group of people applying for international protection in the EU + between July 2021 and December 2022 [8]. With prolonged armed conflict remaining the main driver of the Somali conflict, which is one of the longest conflicts in human history and in its current phase started since 2009 [9], movement to travel abroad will remain a reality in Somalia.

For many female Somali migrants, their first encounter with health care systems in their host countries occurs during pregnancy and childbirth [10]. During this encounter, the parturient and family get acquainted with health personnel as well as the practices around maternal health service in the host country [11]. However, despite the availability of these improved maternal services, migrants often refrain from or have difficulty in accessing care [12]. Somali migrant women have been found to avoid obstetric interventions by deliberately evading prenatal care, switching healthcare facilities and/or providers, delaying hospital arrival during labour, and declining medical treatment [13]. Another study reported that Somali migrants continue to adhere to their cultural practices during pregnancy, exemplified by deliberately reducing food intake to ensure small foetus sizes aimed at avoiding caesarean section (CS), due to the belief that safe delivery is per ‘vaginal’ [12]. Female Somali migrants have a more difficult experience adjusting in their host countries, regarding their navigation of the healthcare systems and maintenance of their health and wellbeing [14]. These different circumstances and practices can increase the risk of poor maternal and perinatal outcomes [15, 16].

There is a lot of primary research that presents data on pregnancy outcomes of Somali migrant women compared to other women living in host countries, however, their results are mixed [15, 17–21]. A 2022 systematic review of pregnancy outcomes of women from conflict-affected countries including Somalia focused on perinatal and neonatal outcomes but not maternal outcomes [22]. An older meta-analysis of routinely collected data that assessed maternal and perinatal outcomes from six countries was published far back in 2008 [15]. Considering this state of the evidence base, the ongoing conflict and recent extreme climate shocks which will potentiate more displacement and migration in Somalia [23], an understanding of adverse pregnancy outcomes of Somali women is needed. Therefore, our objective in this systematic review and meta-analysis is to synthesize available evidence on adverse maternal and perinatal outcomes comparing Somali migrant populations and their host populations, to support evidence-based decisions regarding suitable interventions for Somali migrants in host countries.

## Methods

### Study design

We conducted this systematic review following the updated Preferred Reporting Items for Systematic Reviews and Meta-Analyses (PRISMA) guidelines published in 2020 [24]. The checklist depicting how this review aligns with the PRISMA approach is presented in Additional file 1. The summary of the study design is presented below with the detailed protocol that describes the conduct of the review has been published in the Open Science Framework repository [25].

### Definition of key terms

Some recurring key terms as used in the context of this article to reflect the geographical and population scope of the review are defined in Table 1.

**Table 1:** Definition of key terms

No	Key term	Definition used in this review
1	Global North	Economically advanced countries in Europe, Australia, North America, New Zealand, and East Asia. They include the USA, Canada, European countries, Cyprus, Singapore, Japan, Taiwan, South Korea, New Zealand, Australia, and Israel
2	Migrant	Broadly refers to an individual identified as a migrant, immigrant, asylum seeker or refugee
3	Native/non-migrant	Nationals to whom the term migrant has not applied at any point in their lifetime or generational tree
4	Host country	The country receiving the migrants

### Search strategy

We systematically searched electronic bibliographic databases (PubMed, Scopus, Directory of Open Access Journal and CINAHL Plus) for relevant articles. For the literature search to find these articles, we adapted different keyword combinations to suit each database [Additional file 2]. We selected these databases for their completeness in health-related research areas. We did not apply any date or language restrictions to maximise the scope of our article retrieval. No limit was placed on the start date. However, the search was closed on 30th June 2024 to allow us to proceed with the analysis.

We incorporated Medical Subject Headings in the combinations to narrow our search results to the most relevant articles. The general structure of the search terms combination was informed by the population-intervention-comparison-outcome (PICO) framework, with keywords identified based on our primary outcomes of interest. Population (Somali* OR Migrant* OR immigrant* OR refugee OR asylum seeker), Intervention (maternal* OR antenatal* OR prenatal* OR postnatal* OR delivery OR childbirth OR “obstetric care”), Comparison (same as intervention for native-born pregnant women in host countries in the Global North)[26], and Outcome (“maternal outcome" OR “perinatal outcome” OR "pregnancy outcome"). We combined the terms using Boolean operators in this format ‘(population) AND (intervention) AND (outcome)’. We also reviewed the reference lists of eligible articles for any relevant papers that may have been missed. Finally, we utilised Google Scholar to broaden the scope of our search.

### Eligibility criteria

Using the predefined criteria below, we included primary research that i) reported data on at least one maternal or perinatal outcome among pregnant Somali migrant women, ii) compared outcomes amongst Somali migrant women who migrated to host countries situated in the Global North with those of pregnant women native to those countries, and iii) had their full text available. However, we excluded articles that i) combined results for Somalis and other ethnic groups from which Somali migrant data could not be specifically identified, ii) did not have a comparison group, or iii) were secondary research on any of the primary outcomes of interest.

### Screening and identification of studies for the review

Two independent reviewers (MS and IO) screened the titles and abstracts of the retrieved articles to identify potentially relevant studies. Final eligibility of the retrieved papers was discussed based on stated eligibility criteria and papers were only included if there was agreement. Discrepancies were resolved by discussion between the two reviewers and, if there was no consensus, by discussing with the senior author (AB-T). Multiple publications of the same study and irrelevant articles were identified and excluded. Studies that met the inclusion criteria were subsequently retrieved, and the bibliographic details stored in the automatic reference manager, Mendeley Reference Manager v. 2.110.2 (Elsevier, Amsterdam, Netherlands) for ease of access and in-text citations.

### Quality assessment

We used the 22-item Strengthening the Reporting of Observational Studies in Epidemiology (STROBE) checklist for assessing the quality of reporting in the included studies [27]. We scored studies based on how many of the 22 items from the checklist were adequately reported. For each satisfied criterion, a score of “1” was given or “0” if the requirements were not met. Studies with quality scores of 16 and above as high quality, 12–15 moderate quality, and below 12 as low quality. When the criterion was not applicable to the article, we marked it as “NA”.

### Data extraction

MS and IO managed the extraction of data from the retrieved papers using a standardised data extraction form in Microsoft Excel v.16.72 (Microsoft Corporation, Redmond, USA). For each included study, we extracted data on authors, publication year, aim, objectives, study design, period of the collected data, study country/region, scale of study (facility-based or population-wide), characteristics of sample population, time frame, maternal and perinatal outcomes, including the number, prevalence, or reported odds ratio (OR) of adverse pregnancy events amongst Somali migrant women compared to host population including the 95% confidence interval (95% CI). Where available, we also extracted the *p*-value to depict the strength of any observed association. Data were extracted for both Somali migrant women and native-born women, respectively. Where studies investigated and presented data for other migrant groups, we only utilised data on Somali- and native women. To prevent errors in the data extraction and entry, we assessed data accuracy by cross-checking the entry made in the data extraction sheet with the published text in each article immediately after the original data extraction process.

### Data synthesis and analysis

For the data synthesis, results were summarised and presented in a descriptive format, using tables and figures to describe the characteristics of the included papers, and results explicitly relevant to pregnant Somali migrant women and host population women. We identified studies that reported perinatal outcomes into preterm birth, small for gestational age (SGA), low birth weight (LBW), macrosomia, large for gestational age (LGA), and post-term and presented pooled prevalence of these outcomes independently. In addition, we aligned with the push to adopt a new classification framework that groups preterm birth, SGA, and LBW together under the broad term of the small vulnerable newborn (SVN) recognising its potential programming for action [28]. For other perinatal outcomes, such as APGAR score < 7 at 5 min, breech presentation, birth trauma, we grouped them into labour and delivery complication and reported perinatal mortality to include stillbirth and early neonatal deaths. Maternal outcomes were grouped into induction of labour, CS separating elective and emergency and ‘all CS’ when primary studies did not separate elective and emergency CS, assisted vaginal delivery, secondary arrest of labour/prolonged second stage of labour, pregnancy-induced hypertension and gestational diabetes mellitus.

We conducted a meta-analysis for each group of outcomes where at least two studies presented relevant data. Only studies reporting odds ratios for outcomes were included, excluding nine studies (two focused on maternal outcomes, seven on perinatal outcomes) that reported relative risks. Some studies provided data for multiple outcomes and multiple host population groups, resulting in overlapping study contributions to the forest plots for the same group of outcomes. Each study was included once for each reported outcome-host population group, allowing us to leverage the available data while maintaining the independence of individual outcomes. For continuous variables violating normality, log transformations were applied, with the civartolerance option adjusted in meta set to accommodate potential asymmetry in confidence intervals. Subgroup analyses were performed by host country. Pooled ORs and their 95% confidence intervals (CIs) were calculated using the Mantel–Haenszel method. A random-effects model was chosen due to anticipated heterogeneity across countries and populations. A *p*-value of 0.05 was considered statistically significant. Heterogeneity was assessed using the I^2^ measure, categorized as low (< 25%), moderate (26–75%), or substantial (76–100%). The τ^2^ statistic was also used to quantify between-study variance with values closer to 1 indicating higher heterogeneity. Potential publication bias was evaluated using Egger's test and funnel plots [29]. A *p* > 0.05 indicated no significant evidence of publication bias. We also conducted a leave-one-out sensitivity analysis to assess the influence of individual studies on the overall result, allowing us to identify potential outliers or influential studies that could significantly skew the overall conclusion if removed from the analysis. All meta-analyses and publication bias assessments were conducted using the metan command in Stata version 18.0 (StataCorp LLC., College Station, USA). In instances in which we had only one study for a specific outcome, we did not conduct a meta-analysis. We also did not conduct a leave-one-out sensitivity analysis in instances in which we had only two studies reporting on the specific outcome.

## Results

### Search results

**Fig. 1:**
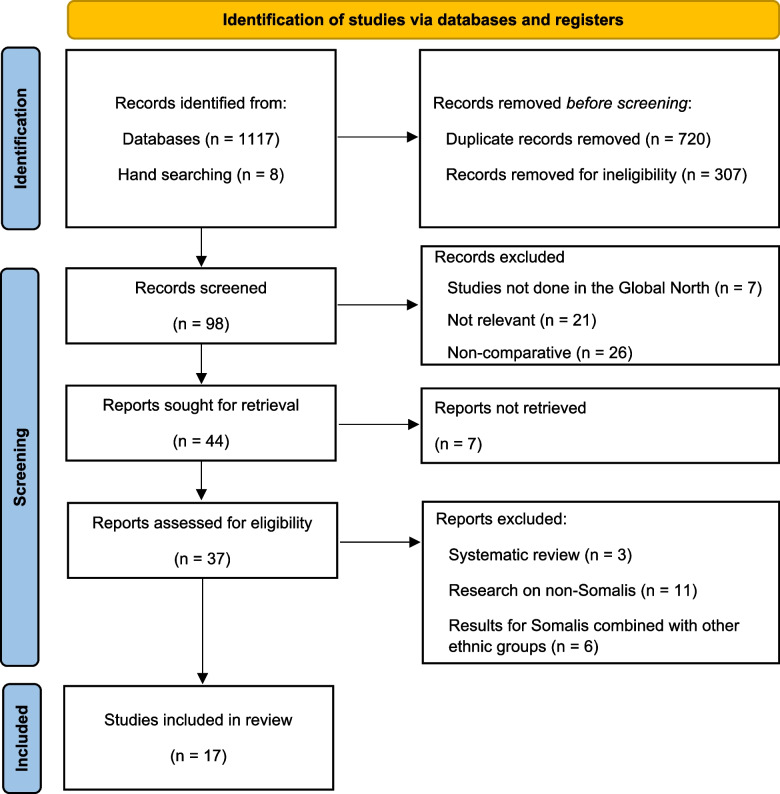
Search results presented using the PRISMA 2020 flow diagram

The electronic search of the literature identified 116 articles. After title and abstract screening to remove duplicates and irrelevant articles, with 98 records screened, of which 44 full-text papers were then retrieved and assessed for eligibility. Of these, 17 articles [10, 11, 15–17, 19–21, 30–38] met the eligibility criteria and were included in this review [Fig. 1].

### Characteristics of included studies

Table 2 provides a summary of the characteristics of the included studies.

**Table 2:** Characteristics of included studies

S/No	Author, Year	Region	Country	Scale/Specific setting	Time frame	Sample size and nationalities	Data source
1	Pedersen et al. 2012 [36]	Europe	Denmark	National	1978–2007	8 555 Somali-born and 1 557 944 Danish women	The Danish Medical Birth Registry, the Danish Civil Registration System, and the Integrated Database for Labour Market Research
2	Rasmussen et al. 2019 [19]	Europe	Denmark	National	2004–2015	953 Somali and 2699 Danish	The Danish Medical Birth Registry
3	Malin and Gissler 2009 [11]	Europe	Finland	National	1999–2001	817 Somali women and 158 469 Finnish	The Finnish Medical Birth Register
4	Bastola, K. et al. 2020 [20]	Europe	Finland	National	2004–2014	584 Somali and 243 Finnish	The National Medical Birth Register and the Hospital Discharge Register
5	Bakken et al. 2015 [30]	Europe	Norway	Baerum hospital, Oslo	2006–2010	278 Somali and 6826 Norwegian	The Medical Birth Registry of Norway
6	Naimy et al. 2013 [32]	Europe	Norway	National	1986–2005	5 410 Somali and 1 062 744 Norwegian	The Medical Birth Registry of Norway and the Central Person Registry of Norway
7	Vangen et al. 2002 [33]	Europe	Norway	National	1986–1998	1 733 Somali and 702 192 Norwegian	The Medical Birth Registry of Norway
8	Akselsson et al. 2020 [31]	Europe	Sweden	Stockholm maternities	2016–2018	278 Somali and 6826 Norwegian	64 Maternity clinics in Stockholm
9	Råssjö et al. 2013 [17]	Europe	Sweden	Hospitals in Stockholm	2001–2009	180 Somali and 507 Swedish	Manual search of labour ward logbooks
10	Belihu et al. 2016 [16]	Oceania	Australia	Sub-national: Victoria	1999–2007	1861 Somali and 427 755 Australians	The Victorian Perinatal Data Collection
11	Flanagan and Mann 2020 [37]	North America	USA	Sub-national: Vermont	2009–2016	190 Somali and 248 control group	Electronic health record from the University of Vermont Medical centre
12	Araneta et al. 2020 [35]	North America	USA	San Diego County	2007–2012	1 095 Somali and 152 078 women of diverse races and ethnicities	Birth cohort database maintained by the California Office of State-wide Health Planning and Development
13	Oliver et al. 2018 [21]	North America	USA	Ohio	2000–2015	8 480 Somali and 314 024 women of other races	Not reported
14	Contag et al. 2021 [34]	North America	USA	Minnesota	2011–2017	13 503 Somali and 65 258 women of diverse races and ethnicities	Minnesota Department of Health and the University of Minnesota
15	Agunwamba et al. 2021 [38]	North America	USA	Olmsted County, Minnesota	2009–2014	296 Somali women and 298 non-Somali women	The Rochester Epidemiology Project
16	Johnson et al. 2005 [10]	North America	USA	Washington state	1993–2001	579 Somali and 4 838 women of other races	The Birth Events Records Database
17	Small et al. 2008 [15]	Mixed	Australia, Belgium, Canada, Finland, Norway, Sweden	National	1997–2004	10 431 Somali and 2 168 891 women from Australia, Belgium, Canada, Finland, Norway, and Sweden	Medical birth registers of all included countries

Of all, 13 of the included studies were published after the start of the ongoing phase of the Somali crisis [16, 17, 19–21, 30–32, 34–38]. The other four studies were published before the current phase [10, 11, 15, 33]. Nine of the 17 included studies (52%) were retrospective cohort studies [10, 11, 19–21, 34–36, 38] with the remaining eight being made up of three cross-sectional studies (18%) [16, 32, 33], three case–control (18%) [17, 30, 31], one prospective cohort (6%) [19], and one primary meta-analysis (6%) [15]. Ten of the 17 studies (58%) drew data on pregnant women from national or sub-national population-based health and birth registers [10, 11, 15, 16, 19, 30, 32, 33, 35, 36] while four were based on hospital records (24%) [17, 20, 31, 37] and the remaining three from other databases (18%) [21, 34, 38]. Nine of the included studies were mostly conducted in Europe (53%), including three from Norway (18%) [30, 32, 33], two (12%) each from Denmark (12%) [19, 36], Finland (12%) [11, 20], and Sweden (12%) [17, 31]. Six (35%) studies were conducted in USA (35%) [10, 21, 34, 35, 37, 38], one in Australia (6%) [16] and another collected and combined data from six countries, including Canada, Australia, Sweden, Finland, Norway, and Belgium (6%) [15]. Two of the included studies (12%), both published in the USA, considered multiple native women across diverse races [10, 34]. Nine studies investigated outcomes for other migrant groups part from Somali women [16, 19–21, 30–32, 35, 36]. Across all included studies, pregnancy-related data on 55 119 Somali migrant women and 5 190 459 women of native origin from 1978 to 2018 were included for analysis [Table 2].

### Quality of the included studies

All 17 articles included in the review were assessed as being of high quality based on the STROBE Checklist quality assessment [10, 11, 15–17, 19–21, 30–38]. The least scoring criteria across included articles were description of any efforts to address potential sources of bias and description of any sensitivity analysis [Additional file 6].

### Results of the meta-analysis

Details of studies included in the meta-analysis are available in the data extraction sheet, including the type of outcome(s) reported, the outcome classification, host country, OR and 95% CI [Additional file 4].

### Maternal outcomes

#### Emergency CS

**Fig. 2:**
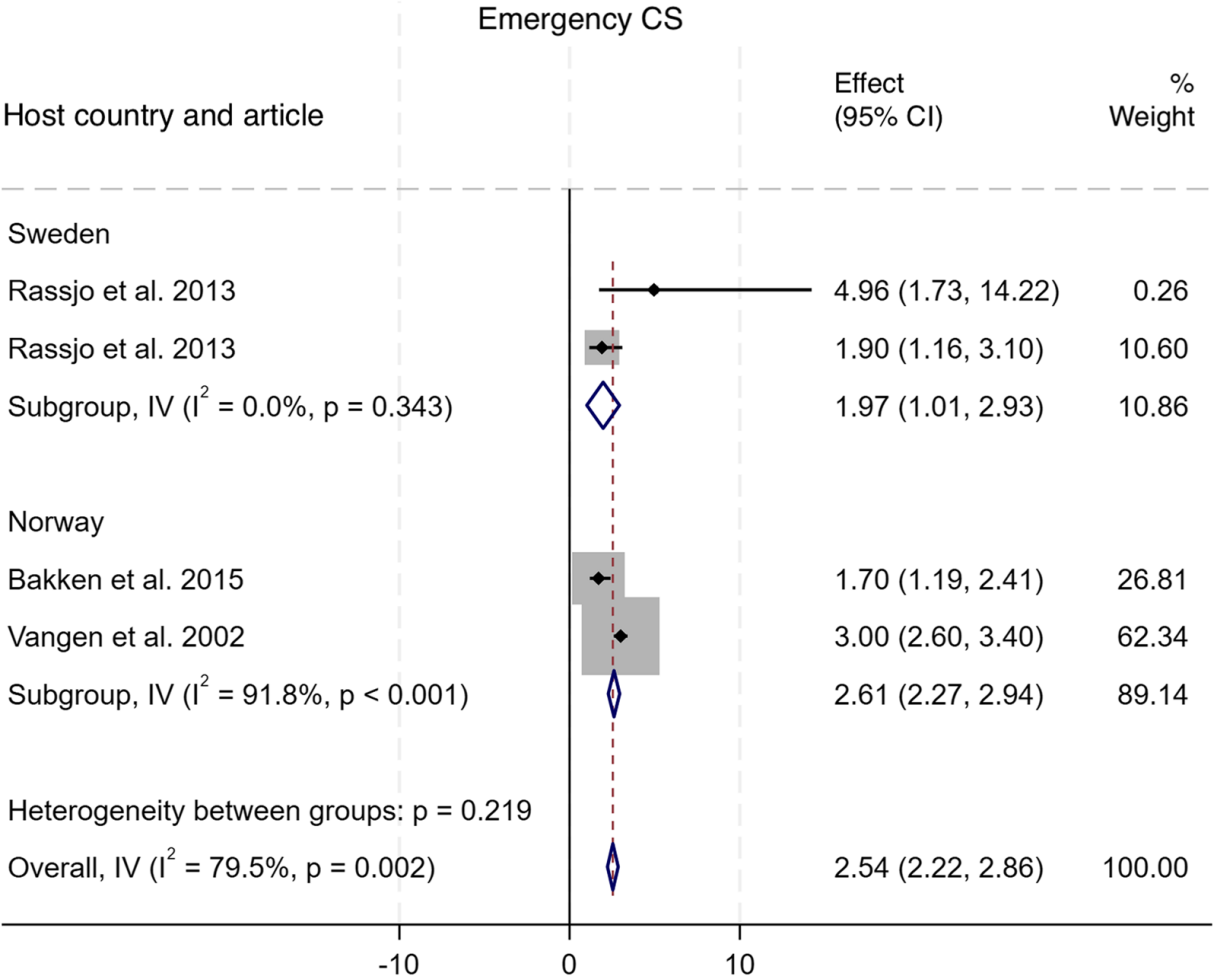
Forest plot for emergency caesarean section

As shown in Fig. 2, three studies compared the incidence of emergency CS among Somali migrants with two host population groups in two countries [17, 30, 33]. The pooled analysis indicated a statistically significant increase in the odds of emergency CS among Somali migrants compared to the host populations, with an OR of 2.54 (95% CI: 2.22–2.86), I^2^ = 79.5%, τ^2^ = 0.57. Specifically, Somali migrant women had significantly higher odds of undergoing emergency CS compared to their host country counterparts in Sweden (OR = 1.97, 95% CI: 1.01–2.93) and Norway (OR = 2.61, 95% CI: 2.27–2.94). Subgroup analysis suggested that host country population groups contributed significantly to the observed heterogeneity, with studies from Sweden (subgroup I^2^ = 91.8%) being the primary driver. Funnel plot asymmetry testing showed no significant evidence of publication bias (*p* = 0.58; see Additional file 5 for funnel plots). Egger’s test for small-study effects also showed no evidence of publication bias (β = 0.95, SE = 1.649, z = 0.58, *p* = 0.5649). There were no significant changes in the overall effect size when studies were singly omitted (see Additional file 6 for the leave-one-out meta-analysis).

#### Elective CS

Only one study reported elective CS, with Somali women having lower odds of having elective CS (OR 0.69, 95% CI 0.35–1.35) [17]. Given we had just one study for this outcome, we could not conduct a funnel plot asymmetry testing.

#### All CS (non-disaggregated)

**Fig. 3:**
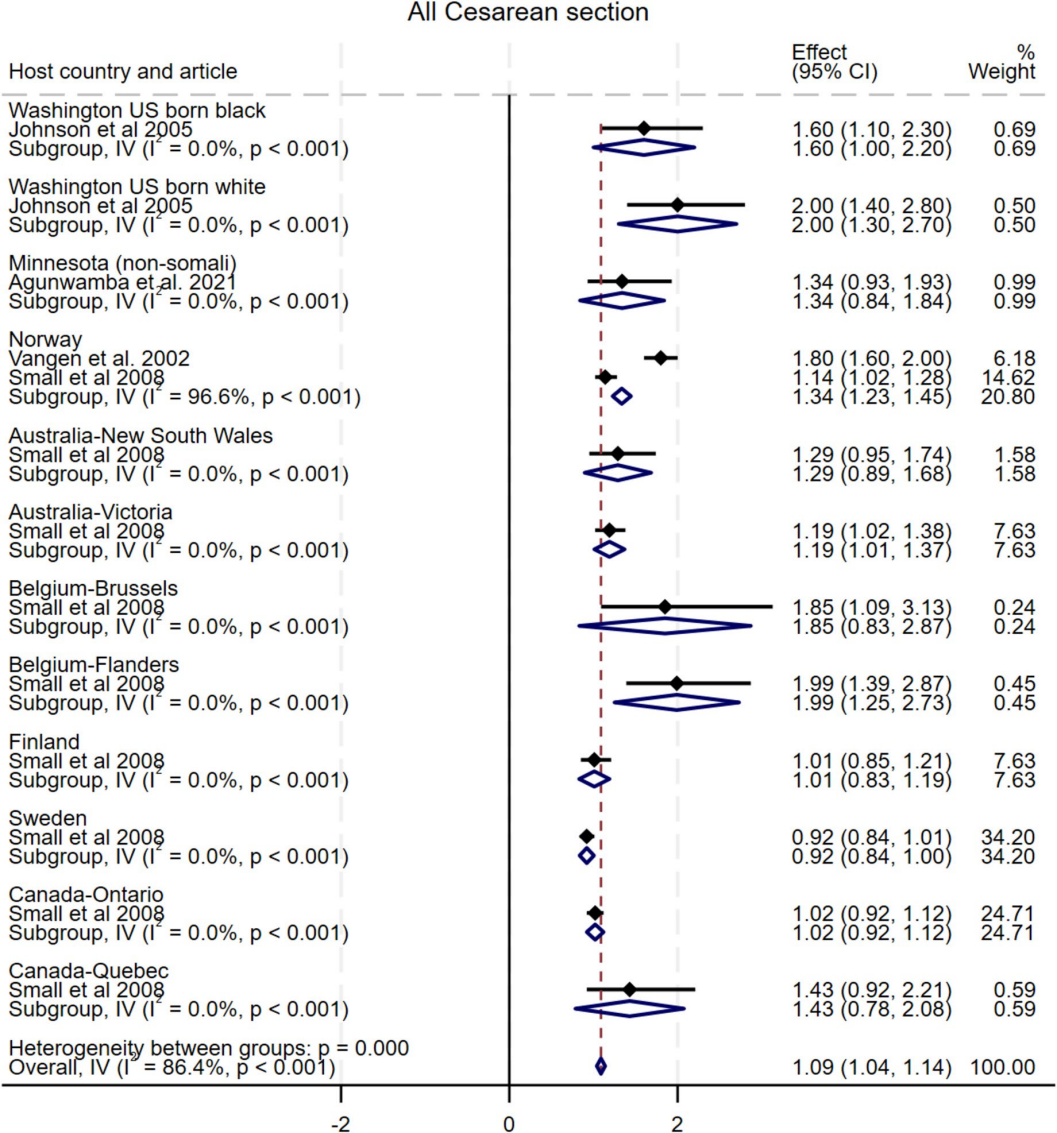
Forest plot for all caesarean section

As shown in Fig. 3, four studies compared the incidence of total non-elective CS among Somali migrants with twelve host population groups in seven countries [10, 15, 33, 38]. The pooled analysis indicated a statistically significant increase in the odds of CS among Somali migrants compared to the host populations, with a pooled OR of 1.09 (95% CI: 1.04–1.14), I^2^ = 86.4%, τ^2^ = 0.06. Funnel plot asymmetry testing showed evidence of publication bias (*p* = 0.00; see Additional file 5 for funnel plots). Egger’s test for small-study effects also showed no evidence of publication bias (β = 0.19, SE = 0.91, z = 0.21, *p* = 0.84). There were no significant changes in the overall effect size when studies were singly omitted (see Additional file 6 for leave-one-out meta-analysis).

#### Assisted instrumental delivery

**Fig. 4:**
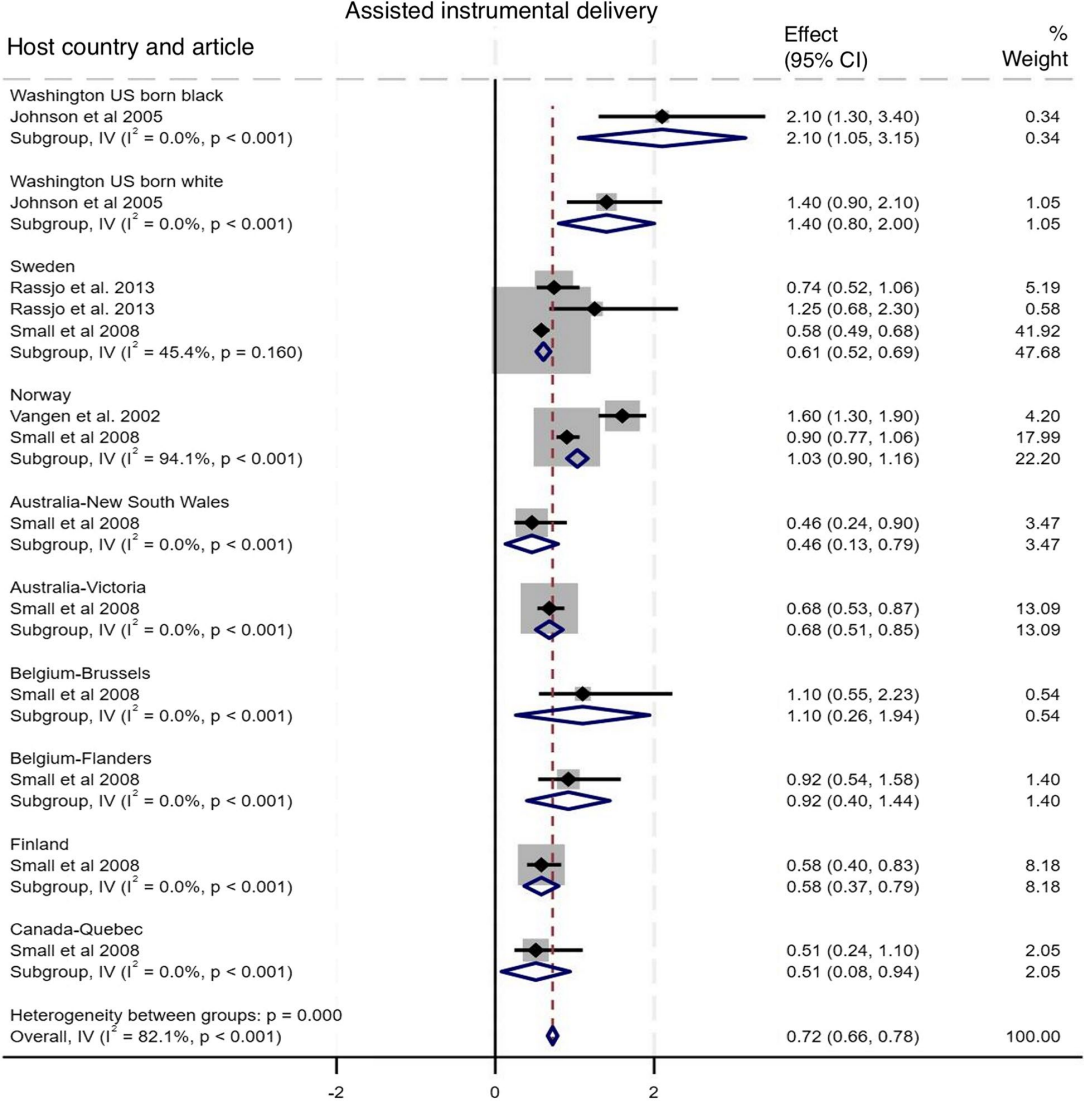
Forest plot for assisted instrumental delivery

As shown in Fig. 4, four studies compared the incidence of assisted instrumental deliveries among Somali migrants with ten host population groups across seven countries [10, 15, 17, 33]. The pooled analysis indicated a statistically significant lower odds of assisted instrumental deliveries among Somali migrants compared to the host populations, with an OR of 0.72 (95% CI: 0.66–0.78), I^2^ = 82.1%, τ^2^ = 0.07. While Somali migrants had higher odds of assisted instrumental delivery compared to Washington US born black (OR 2.10, 95%CI 1.05–3.15), the odds were lower compared to host country counterparts in Sweden (OR 0.61, 95%CI 0.52–0.69), Australia New South Wales (OR 0.46, 95%CI 0.13–0.79, Australia Victoria (OR 0.68, 95% CI 0.51–0.85, Finland (OR 0.58, 5%CI 0.37–0.79,and Canada Quebec (OR 0.51, 95% CI 0.08–0.94). Subgroup analysis suggested that host country population groups contributed significantly to the observed heterogeneity, with studies from Sweden (subgroup I^2^ = 94.1%) and Norway (subgroup I^2^ = 45.4%) being the primary drivers. Funnel plot asymmetry testing showed no significant evidence of publication bias (*p* = 0.89; see Additional file 5 for funnel plots). Egger’s test for small-study effects also showed no evidence of publication bias (β = 0.00, SE = 0.563, z = 0.00, *p* = 1.000). There was a significant change in the overall effect size when we omitted the Small et al. 2008 study [15], with OR of 1.19 (95% CI: 1.00–1.37), I^2^ = 81.2%, τ^2^ = 0.06. There were no significant changes in the overall effect size when other studies were singly omitted (see Additional file 6 for leave-one-out meta-analysis).

#### Non-progressing and induced labour

**Fig. 5:**
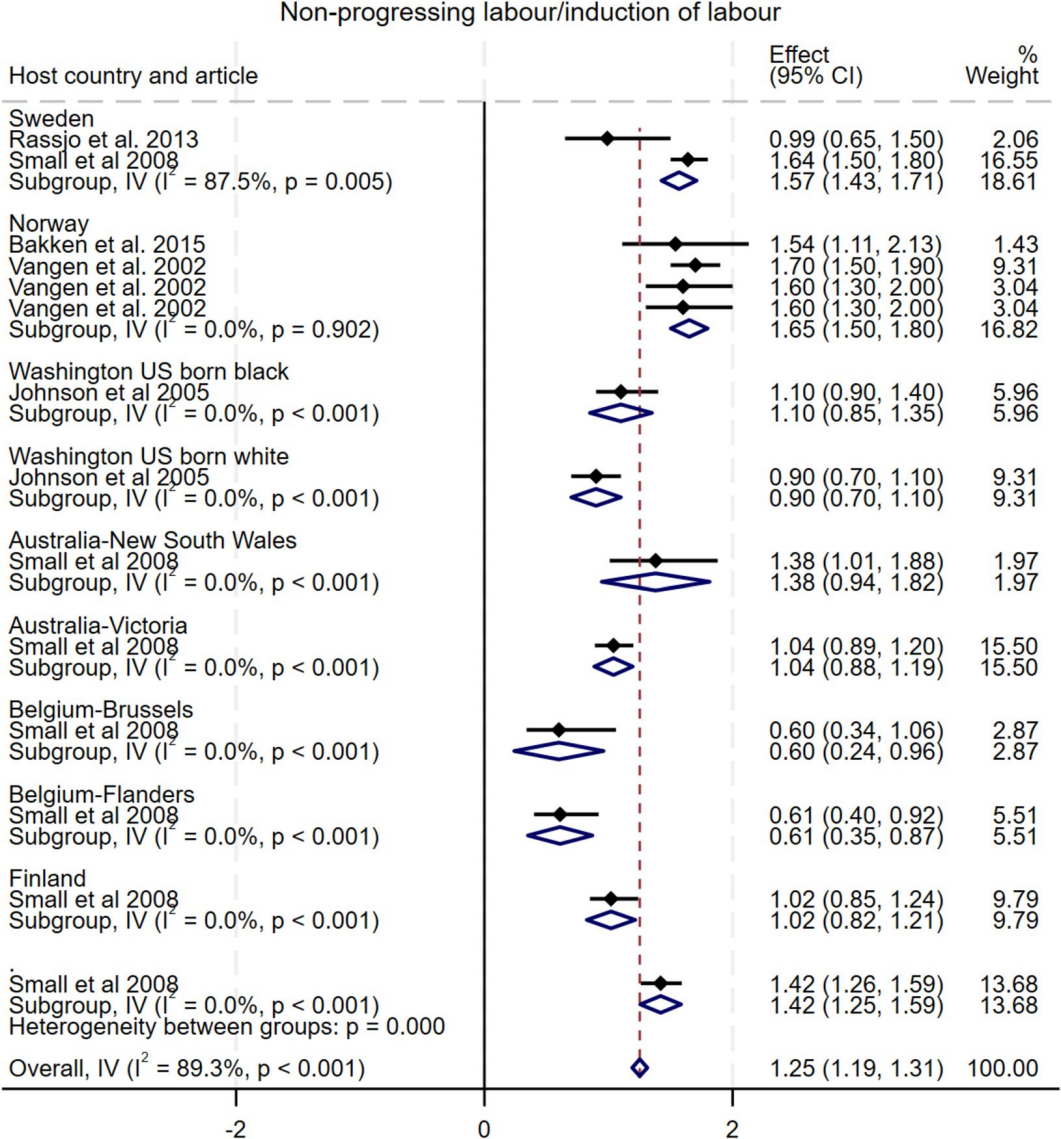
Forest plot for non-progressing and induced labour

As shown in Fig. 5, five studies compared the incidence of non-progressing and induced labour among Somali migrant women with nine host population groups across six countries [10, 15, 17, 30, 33]. The pooled analysis indicated a statistically significant increase in the odds of non-progressing/induced labour among Somali migrant women compared to the host population groups, with an OR of 1.25 (95% CI: 1.19–1.31), I^2^ = 89.3%, τ^2^ = 0.12. Specifically, Somali migrants had significantly higher odds of non-progressing/induced labour compared to their Swedish and Norwegian counterparts (OR = 1.57, 95% CI: 1.43–1.71; OR 1.55, 95% CI 1.44–1.66, respectively), and lower odds compared to Belgium-Flanders (OR = 0.61, 95% CI: 0.35–0.87). Subgroup analysis show that observed heterogeneity was driven by studies from Sweden (subgroup I^2^ = 87.5%) and Norway (subgroup I^2^ = 14.9%). Funnel plot asymmetry testing showed significant evidence of publication bias (*p* < 0.001; see Additional file 5 for funnel plots). Egger’s test for small-study effects indicated evidence of publication bias (β = −3.28, SE = 1.14, z = −2.87, *p* < 0.01). There were no significant changes in the overall effect size when studies were singly omitted (see Additional file 6 for leave-one-out meta-analysis).

#### Post-partum depression

One study compared incidence of post-partum depression in Somali migrants to host country women in Minnesota USA, with Somali women having lower odds of experiencing post-partum depression (OR 0.27, 95% CI 0.12–0.63) [38].

#### Pregnancy induced hypertension, gestational diabetes

One study reported no significant difference in the incidence of pregnancy induced hypertension and gestational diabetes in Somali migrants compared to their host country counterparts in Finland (OR 1,27, 95% CI 0.81–2; OR 0.69, 95% CI 0.35–1.35) [20].

#### Intra-partum tear/post-partum haemorrhage

One study compared the incidence of Intra-partum tear/post-partum haemorrhage among Somali migrants with one host population group in one country (Norway) [33]. The analysis showed no difference in the odds of intra-partum tear (OR 1.2, 95% CI 0.9–1.6) or post-partum haemorrhage (OR 1.3, 95% CI 1–1.6) among Somali migrants compared to their Norwegian counterparts.

### Perinatal outcomes

#### Small vulnerable newborn

**Fig. 6:**
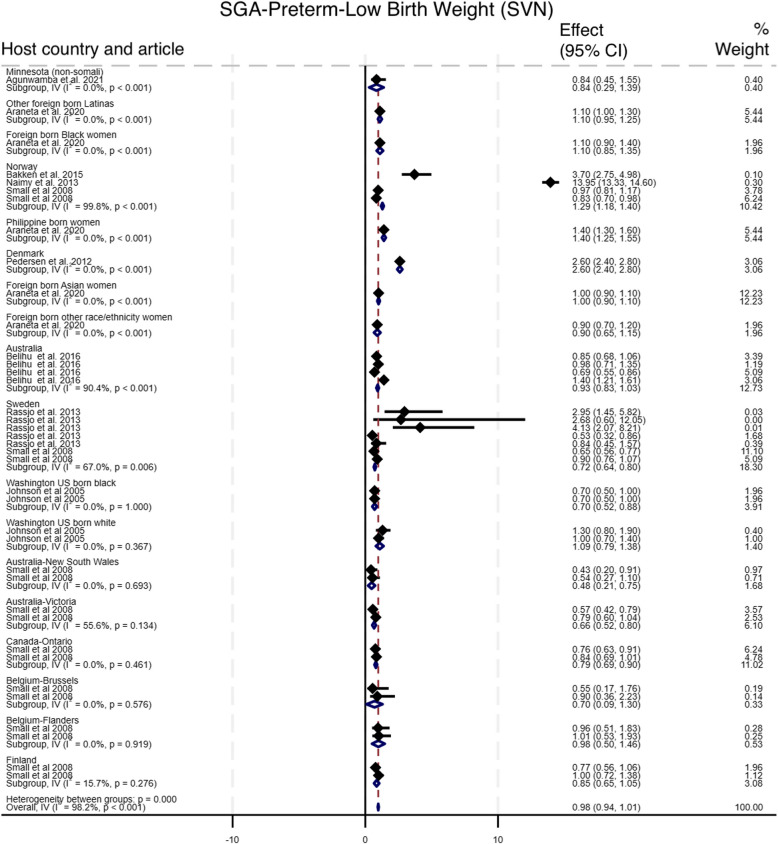
Forest plot for small vulnerable newborn

**Fig. 7:**
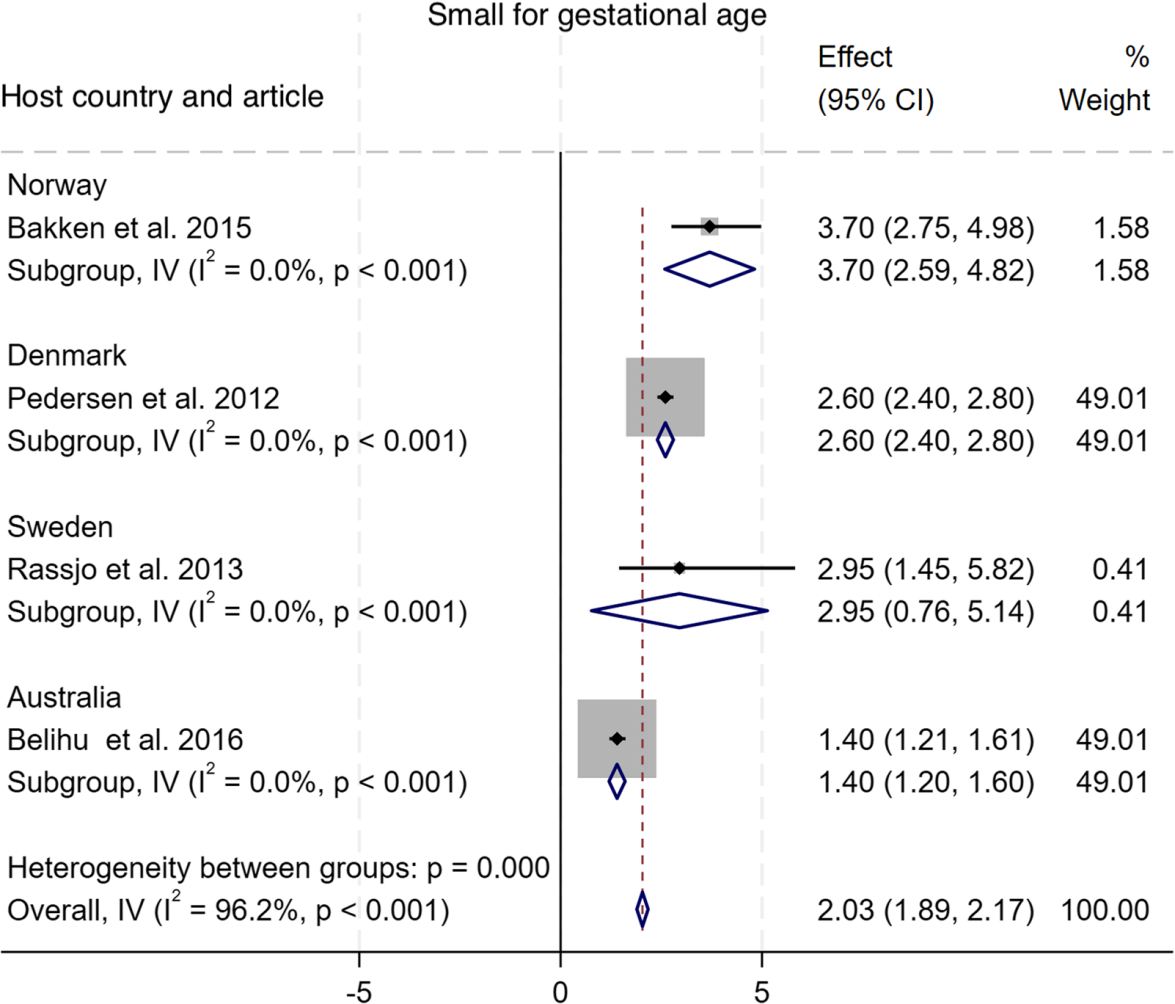
Forest plot for small for gestational age

**Fig. 8:**
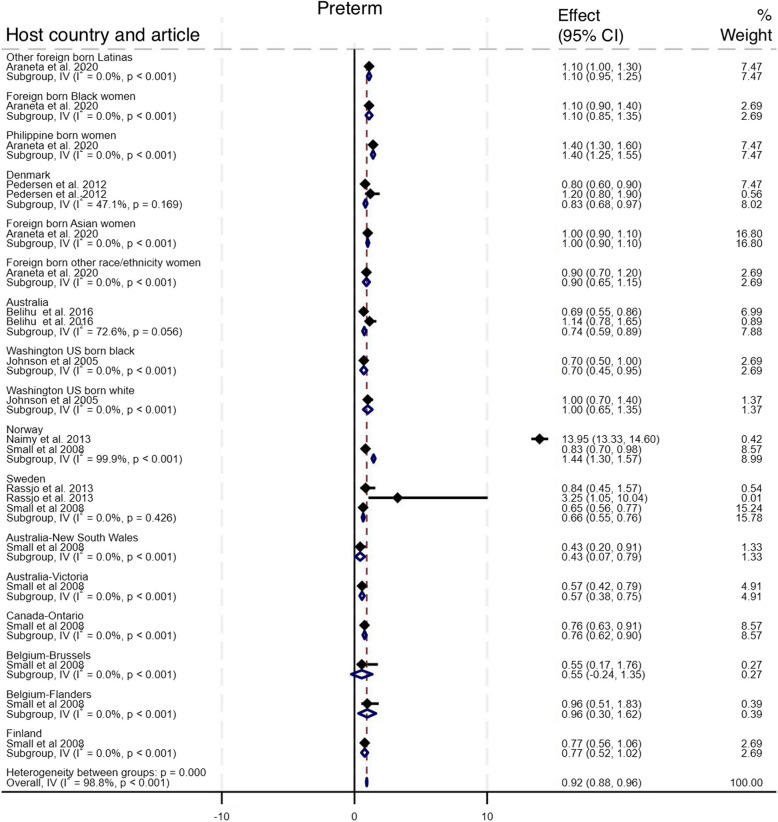
Forest plot for preterm birth

**Fig. 9:**
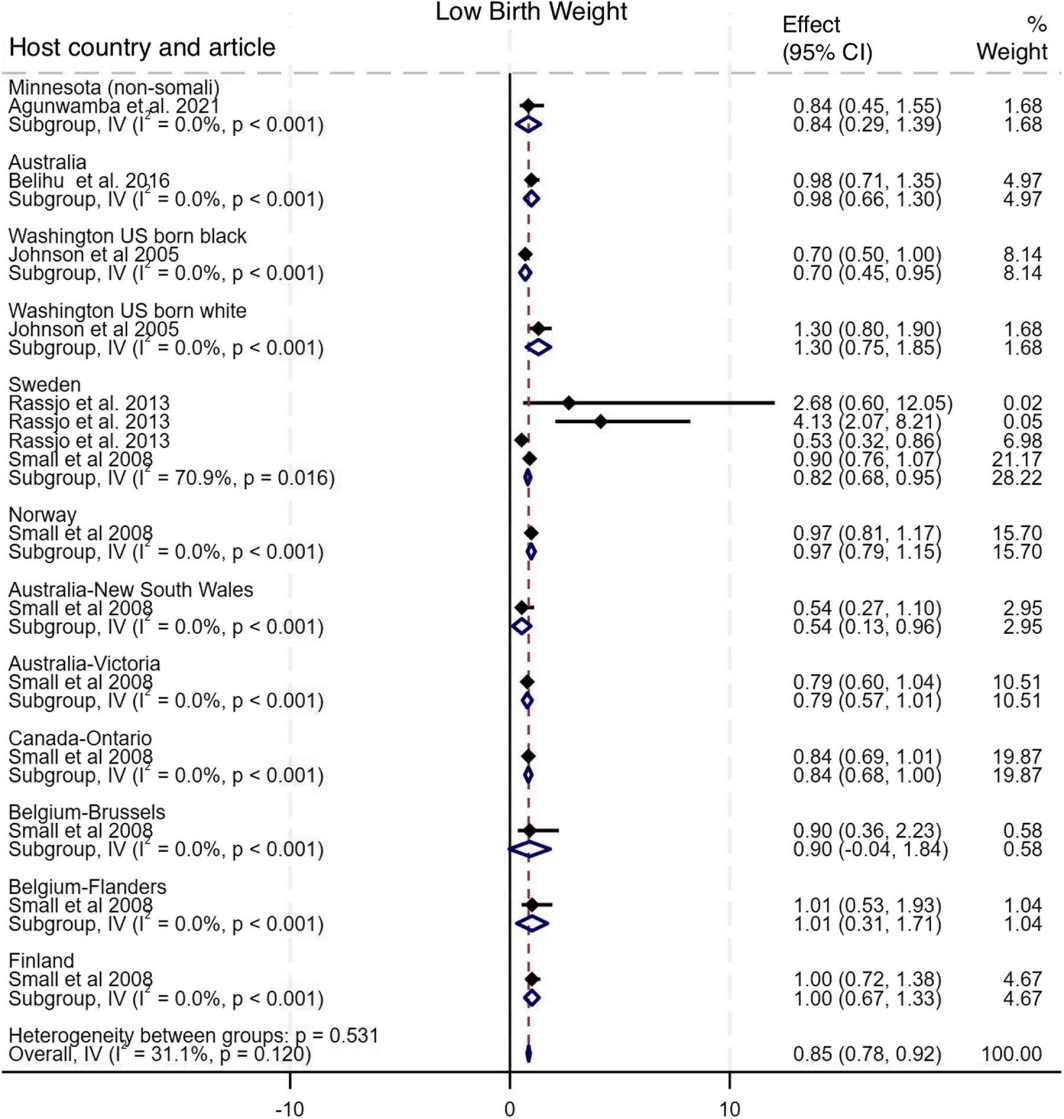
Forest plot for low birth weight

As shown in Fig. 6, nine studies compared the incidence of adverse outcomes classified as SVN among newborn of Somali migrant women with eighteen host population groups across eight countries [10, 15–17, 30, 32, 35, 36, 38]. The pooled analysis showed no difference in the odds of SVN among babies of Somali migrant women compared with host population groups (Pooled OR 0.98, 95% CI 0.94–1.01, I^2^ = 98.0%, τ^2^ = 0.66). Sub-group analysis of SVN group components (SGA, preterm, LBW) showed that Somali migrants had higher odds of SGA (OR 2.03, 95% CI 1.89–2.17, I^2^ = 96.2%, τ^2^ = 0.74), lower odds of preterm (OR 0.92, 95% CI 0.88–0.96, I^2^ = 98.8%, τ^2^ = 0.83), and LBW (OR 0.85, 95% CI 0.78–0.92, I^2^ = 31.1%, τ^2^ = 0.07) [Figs. 7, 8, 9]. Funnel plot asymmetry testing showed evidence of no publication bias (*p* = 0.28, 0.36 and 0.07 respectively; see Additional file 5 for funnel plots). Egger’s test for small-study effects corroborated suggestion of no evidence of publication bias (β = 1.39, *p* = 0.52), (β = 1.01, *p* = 0.17), (β = −2.5, *p* = 0.06) respectively. There was a significant change in the overall effect size when we omitted the Small et al. 2008 study [15], with OR of 1.18 (95% CI: 1.13–1.23), I^2^ = 98.7%, τ^2^ = 0.05. There were no significant changes in the overall effect size when other studies were singly omitted (see Additional file 6 for leave-one-out meta-analysis).

#### Macrosomia

**Fig. 10:**
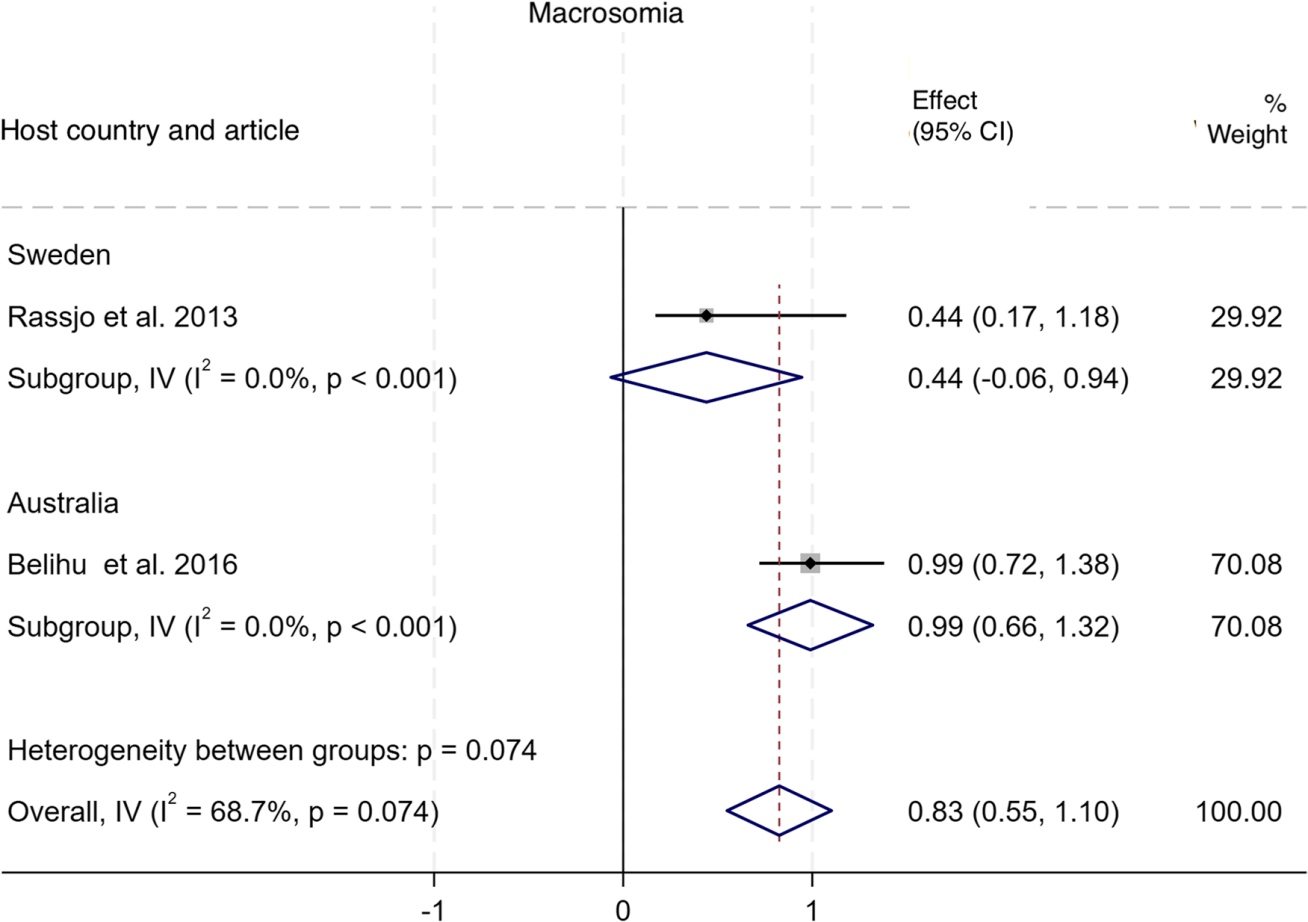
Forest plot for macrosomia

As shown in Fig. 10, two studies compared the incidence of adverse outcomes related to macrosomia among babies of Somali migrant women with two host population groups in two countries [16, 17]. The pooled analysis shows no difference in the odds of having macrosomia babies among Somali migrants compared to the host populations, with an OR of 0.83 (95% CI: 0.55 – 1.10), I^2^ = 68.7%, τ^2^ = 0.1; See Additional file 5 for funnel plots).

#### Neonatal morbidity

**Fig. 11:**
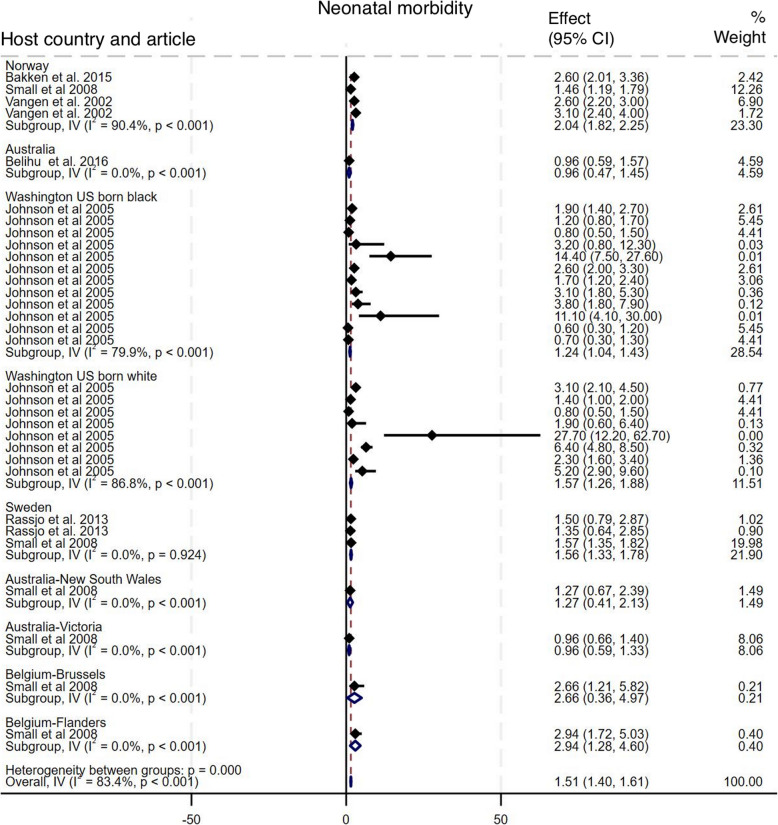
Forest plot for neonatal morbidity

As shown in Fig. 11, six studies compared the incidence of neonatal morbidity among babies of Somali migrant women with eight host population groups across five countries [10, 15–17, 30, 33]. The pooled analysis shows a statistically significant increased odds of neonatal morbidity among babies of Somali migrant women compared to the host populations, with an OR of 1.51 (95% CI: 1.40 – 1.61), I^2^ = 83.4%, τ^2^ = 0.49. Subgroup analysis show that Somali migrant women had significantly higher odds of neonatal morbidity compared to all host population groups except those in Australia and Belgium-Brussels. Funnel plot asymmetry testing showed no significant evidence of publication bias (*p* = 0.29; see Additional file 5 for funnel plots). Egger’s test for small-study effects also indicated no evidence of publication bias (β = 1.14, SE = 1.09, z = 1.04, *p* = 0.28). There were no significant changes in the overall effect size when studies were singly omitted (see Additional file 6 for leave-one-out meta-analysis).

#### Neonatal mortality

**Fig. 12:**
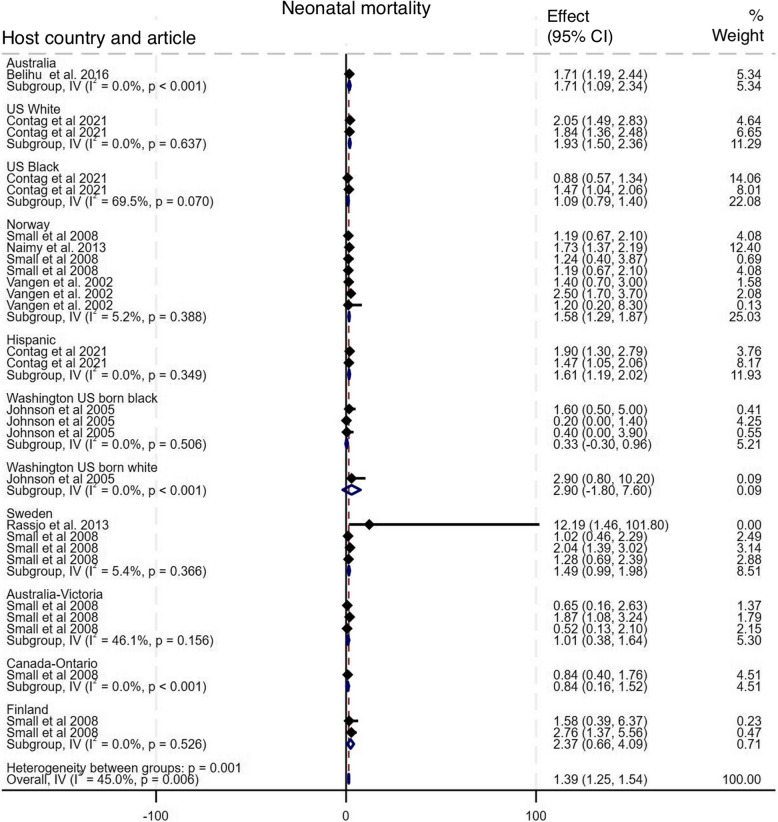
Forest plot for neonatal mortality

fAs shown in Fig. 12, seven studies compared the incidence of neonatal mortality among babies of Somali migrant women with ten host population groups across six countries [10, 15–17, 32–34]. The pooled analysis shows a statistically significant increased odds of neonatal mortality among Somali migrant women compared to the host populations, with an OR of 1.39 (95% CI: 1.25 – 1.54), I^2^ = 45%, τ^2^ = 0.12. Subgroup analysis show that Somali migrant women had significantly higher odds of neonatal mortality compared to host population groups in Norway (OR 1.58, 95% CI 1.29–1.87), Australia (OR 1.71, 95% CI 1.09–2.34), US white (OR 1.93, 95% CI 1.50–2.36), and Hispanic (OR 1.61, 95% CI 1.19–2.02). Funnel plot asymmetry testing showed no significant evidence of publication bias (*p* = 0.66; see Additional file 5 for funnel plots). Egger’s test for small-study effects also indicated no evidence of publication bias (β = −0.49, SE = 0.44, z = −1.11, p = 0.27. There were no significant changes in the overall effect size when studies were singly omitted (see Additional file 6 for leave-one-out meta-analysis).

## Discussion

This systematic review and meta-analysis comprehensively examined adverse maternal and perinatal outcomes among Somali migrant women compared to host country population groups. We retrieved slightly higher number of studies published in European countries (nine) compared to USA (six) and only one in Australia. For the latter country, Australia, the number is surprisingly few, especially considering that a 35% increase in Somali-born people living in the country was reported between 2011 and 2016 based on national census figures [39]. Irrespective of where studies were published, majority (almost 80%) were based on large-scale national or sub-national databases [10, 11, 15, 16, 19, 21, 30, 32–36, 38], which probably contributed to the overall high quality we observed in the included studies.

In terms of findings from our pooled assessment of the maternal outcomes, we found a number of significant outcome disparities between Somali migrant women and their host populations. First, the pooled OR of non-progressing/induced labour was significantly higher amongst Somali migrant women compared to host population groups. The increased likelihood of labour non-progression and induction may also explain the significantly higher odds of CS amongst Somali women. This association was particularly pronounced in comparisons with populations in Sweden and Norway. Our finding varies from that of a systematic review by Behboudi-Gandevani et al. which compared pooled prevalence of maternal and perinatal outcomes between migrants from 12 conflict-zone countries including Somalia and host populations and concluded that there was no significant difference in CS between both groups [22]. Of the studies we included in our review, one primary study in Norway reported higher odds of intra-partum tear and post-partum haemorrhage amongst Somali migrant women compared to host population groups [33]. Put together, it might be that for various reasons leading to delay in accessing care including language and cultural barriers, lack of health insurance, and being circumcised [14, 40] Somali migrant women are having a higher chance of obstetric complications which then means higher risk of giving birth via CS compared to their host population. This explanation is further underscored by the significantly higher odds that we observed when we disaggregated the CS category to specifically highlight emergency CS (pooled OR 2.54 (CI 2.22–2.86)).

However, as per our pooled analysis, Somali women were less likely to give birth via assisted instrumental deliveries. This finding contrasts with the conclusion of the other published systematic review that included migrant women from multiple conflict-affected countries [22]. Given that this result also differed from the higher pooled OR we found for CS in our review, we recognised that a nuanced interpretation was required. As such, we assessed the influence of individual studies on the overall effect size. The leave-one-out meta-analysis showed that the overall effect size only changed, and in the opposite direction, when we omitted the multi-country study by Small et al. 2008 study [15]. A closer examination of individual studies that assessed assisted instrumental deliveries revealed diverging conclusions regarding the direction of association [10, 15, 33]. For example, in Norway, the OR reported by Small et al. differed from that reported by Vangen et. al., with the latter concluding that Somali women had a higher likelihood of assisted instrumental deliveries [15, 33]. Data for Vangen et al.’s paper covered the period 1986 to 1998, pre-dating the Term Breech Trial, which is believed to have influenced the observed increase in planned CS in Norway [41]. A plausible explanation for these variations is that since both assisted instrumental deliveries and CS serve as alternatives to vaginal delivery, the preferred intervention may depend on the clinical judgment of skilled health personnel and prevailing obstetric guidelines at the time. Further, evidence suggests that some Somali women avoid obstetric interventions with a study in Franklin County, Ohio, USA showing that Somali women deliberately changed hospitals and healthcare providers, delayed their arrival at hospital during labour, and even refused care when offered [13]. Separately, the only one study that assessed post-partum depression found that Somali migrant women had significantly lower odds of postpartum depression [38]. This may reflect protective cultural factors, such as strong community support networks, or under-reporting due to cultural stigma surrounding mental health conditions.

For perinatal outcomes, our findings indicate a significantly higher likelihood of post term deliveries among Somali women (OR = 1.80 (95% CI 1.47–2.13)), which also probably contributes to the increased likelihood for induced labour that we found in our meta-analysis. Our effort to aggerate SGA, preterm, and LBW into the widely advocated SVN outcome did not yield a significant association (Pooled OR = 0.97 (95% CI 0.94–1.00)). However, after omitting the Small et al. (2008) study [15], we found a significantly higher likelihood of SVN amongst Somali women compared to host populations (Pooled OR = 1.18 (95% CI 1.13–1.23)). When we analysed the three SVN components separately, we found significantly higher odds for SGA (Pooled OR = 2.03 (95% CI 1.89–2.17)) but lower odds for preterm (Pooled OR = 0.92 (95% CI 0.88–0.96)) and LBW (Pooled OR = 0.87 (CI 0.80–0.94)). Similar patterns have been in studies comparing outcomes amongst migrant women from multiple conflict-affected countries to host population, where higher odds of SGA and lower odds of preterm births were reported [22]. Our leave-one-out analysis showed that preterm reported in Small et al., 2008 paper [15] was the key driver of these diverging conclusions. We opine that the direction of association depends on the specific comparison host population. In a study comparing Somali women with Latinas and Philippine-born women, Somali women had significantly higher odds of preterm births [35]. In contrast, another study comparing Somali women to a predominantly Caucasian population reported significantly lower odds [15]. Indeed, evidence suggests that some underlying factors including a complex interaction between parental and foetal genetics, epigenetics, behavioural, and sociodemographic risk factors underpin differing rates in preterm births observed across racial groups [42]. For example, smoking, a major risk for preterm birth, is negatively perceived within the Somali population [43]. Conversely, evidenced association between maternal nutritional status and SGA [44] may explain the observed association in our meta-analysis. A study in Maine, USA, showed that almost seven out of every 10 recent Somali arrivals reported some level of food insecurity [45], which leads to malnutrition. In addition, while we found no difference in macrosomia > 4.5kg between Somali migrant women and host populations (Pooled OR = 0.83 (CI 0.55–1.10)), the review by Behboudi-Gandevani et al. showed Somali migrant women were less likely to have macrosomic babies compared to host populations [22]. Finally, 5-min Apgar score < 7, stillbirth, and perinatal mortality, were significantly higher amongst Somali women compared with women of the host population – a similar conclusion reached in the earlier systematic review [22]. This might be explained by the predilection for late presentation and the other factors that predispose Somali women to higher likelihood of giving birth via CS [13].

A strength of this systematic review and meta-analysis is its specific focus on a population of interest. By concentrating on Somali migrant women, our study provides valuable insights into the specific maternal health challenges faced by this group, contributing to the growing body of evidence on migrant health disparities. In addition, we conducted a leave-one-out meta-analysis that allowed us to assess the influence of individual studies on the overall effect size and better explain the observed associations. However, there are key limitations that need to be highlighted. First, despite our best efforts, there were some outcomes such as pregnancy-induced hypertension and gestational diabetes, for which we could not find sufficient articles to allow for a meta-analysis. However, we conducted a meta-analysis on outcomes as reasonably as possible [46]. Second, though we were able to provide pooled estimate for emergency CS, we could not do same for elective CS, as many articles did not separate total CS into emergency and elective CS. Third, although funnel plot asymmetry tests did not reveal significant evidence of publication bias for most outcomes, an exception was found for non-progressing/induced labour. This suggests that while the overall results are generally robust, these specific outcomes may be influenced by publication bias. Fourth, substantial heterogeneity, which was expected, was noted across most outcomes, indicating significant variability in healthcare systems, study designs, primary outcomes of interest and definitions of maternal and perinatal outcomes across the included studies. Indeed, host country differences explained some of the observed between-study heterogeneity in our study. For example, subgroup analyses revealed that studies from Sweden and Norway were primary contributors to this heterogeneity in multiple outcomes. Fifth, while the systematic review focuses on Somali women, it does not account for the diversity within the Somali population itself, such as differences in ethnicity, socioeconomic status, length of stay in the host country, or access to healthcare services, all of which can influence outcomes. Finally, the variability in study design, population characteristics, and healthcare practices across countries limits the generalizability of the findings.

### Implications for policy, practice, and future research

The findings of this review have significant implications for policy and clinical practice. The observed disparities in maternal and perinatal outcomes for Somali migrant women underscore the need for supportive and culturally competent healthcare services including prenatal and obstetric care that address the unique needs of migrant populations. This could involve targeted interventions to improve prenatal care access, language services, and culturally tailored health education such as cultural health navigators in Arizona, US [47] and doulas to provide emotional and practical support through the pregnancy [48]. Lower rates of CS and greater satisfaction with care have been reported among doula-supported Somali women [48]. Any such strategies should leverage good experience of Somali women who have engaged with health services, while intently upskilling competence of skilled health personnel to provide respectful, culturally aware, and trauma-informed care to Somali migrant women [49].

For future research, our review situated within the broader literature has clearly shown that women from conflict-affected countries are not necessarily a homogenous group. As such, there is a need for more population-specific research for migrant populations and when lumped together, more nuanced interpretation when comparing outcomes across populations. Additionally, more granular research is needed to differentiate between immigrants, asylum seekers, and refugees to understand their specific needs and outcomes better. Also, the host population comparator really matters in interpreting findings. Such research could help in developing more nuanced and effective interventions and policies. Future research should also explore the impact of different healthcare delivery models on these outcomes, as well as the role of social determinants of health. In addition, it is striking that there is no study on maternal mortality. especially as this is generally commoner amongst migrant women [50].

## Conclusion

Overall, our study highlights significant disparities in maternal and perinatal outcomes between Somali migrant women and host populations. The findings suggest a need for culturally sensitive and supportive healthcare practices and policies that address the specific needs of Somali migrants. Further research should focus on understanding the underlying causes of these disparities and developing interventions to improve outcomes in this population.

## Supplementary Information

Supplementary Material 1

Supplementary Material 2

Supplementary Material 3

Supplementary Material 4

Supplementary Material 5

Supplementary Material 6

## Data Availability

The datasets used for this review are available in the additional files.
