# Supplementary material for: Maternal and perinatal outcomes of Somali migrant women in comparison to host populations in the Global North: a systematic review and meta-analysis
Source: Matern Health Neonatol Perinatol. 2025 Jun 3;11:14. doi: 10.1186/s40748-025-00210-1 (PMC12131341; doi:10.1186/s40748-025-00210-1)
Supplement: Supplementary file 2 — Supplementary Material 2 [file 40748_2025_210_MOESM2_ESM.pdf]

Search strategy

Database	Search terms	Number of hits
DOAJ	(Somali) AND (maternal* OR antenatal* OR prenatal* OR postnatal* OR delivery OR childbirth OR “obstetric care”) AND (“maternal outcome" OR “perinatal outcome” OR "pregnancy outcome")	6
PubMed	(("somali*"[All Fields] OR "migrant*"[All Fields] OR "immigrant*"[All Fields] OR "refugee"[All Fields] OR "asylum seeker"[All Fields]) AND ("maternal*"[All Fields] OR "antenatal*"[All Fields] OR "prenatal*"[All Fields] OR "postnatal*"[All Fields] OR "delivery"[All Fields] OR "childbirth"[All Fields] OR "obstetric care"[All Fields]) AND ("maternal outcome"[All Fields] OR "perinatal outcome"[All Fields] OR "pregnancy outcome"[All Fields])) AND (1000/1/1:2024/6/30[pdat])	363
CINNAHL plus with full text via EBSCOhost	((“Somali*” OR “Migrant*” OR “immigrant*” OR “refugee” OR “asylum seeker”)) AND ((“maternal*” OR “antenatal*” OR “prenatal*” OR “postnatal*” OR “delivery” OR “childbirth” OR “obstetric care”)) AND ((“maternal outcome" OR “perinatal outcome” OR "pregnancy outcome")) <20240630	26
Scopus	(TITLE-ABS-KEY ("Somali*" OR "Migrant*" OR "immigrant*" OR "refugee" OR "asylum seeker") AND TITLE-ABS-KEY ("maternal*" OR "antenatal*" OR "prenatal*" OR "postnatal*" OR "delivery" OR "childbirth" OR "obstetric care") AND TITLE-ABS-KEY ("maternal outcome" OR "perinatal outcome" OR "pregnancy outcome")) PUBYEAR < 2025	712
Total hits		
