# Supplementary material for: Maternal and perinatal outcomes of Somali migrant women in comparison to host populations in the Global North: a systematic review and meta-analysis
Source: Matern Health Neonatol Perinatol. 2025 Jun 3;11:14. doi: 10.1186/s40748-025-00210-1 (PMC12131341; doi:10.1186/s40748-025-00210-1)

Maternal and perinatal outcomes of Somali migrant women in comparison to host populations in the Global North: a systematic review and meta-analysis Said et al. 2025

Funnel_All CS

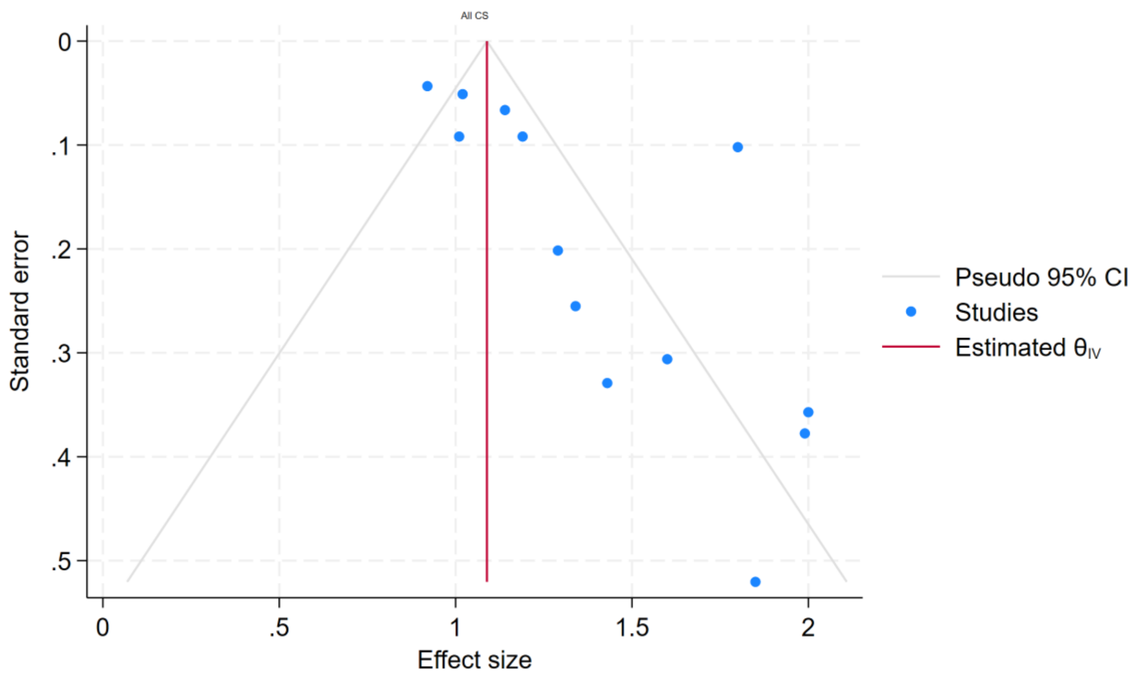

Funnel_Emergency CS

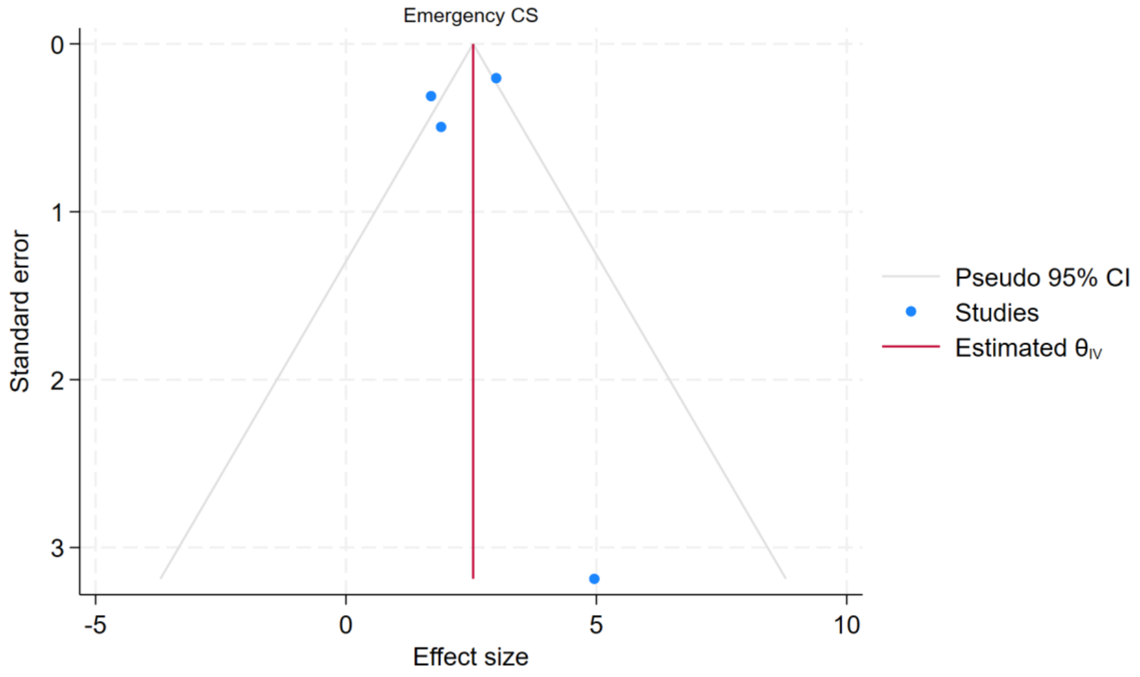

Funnel_low birth weight

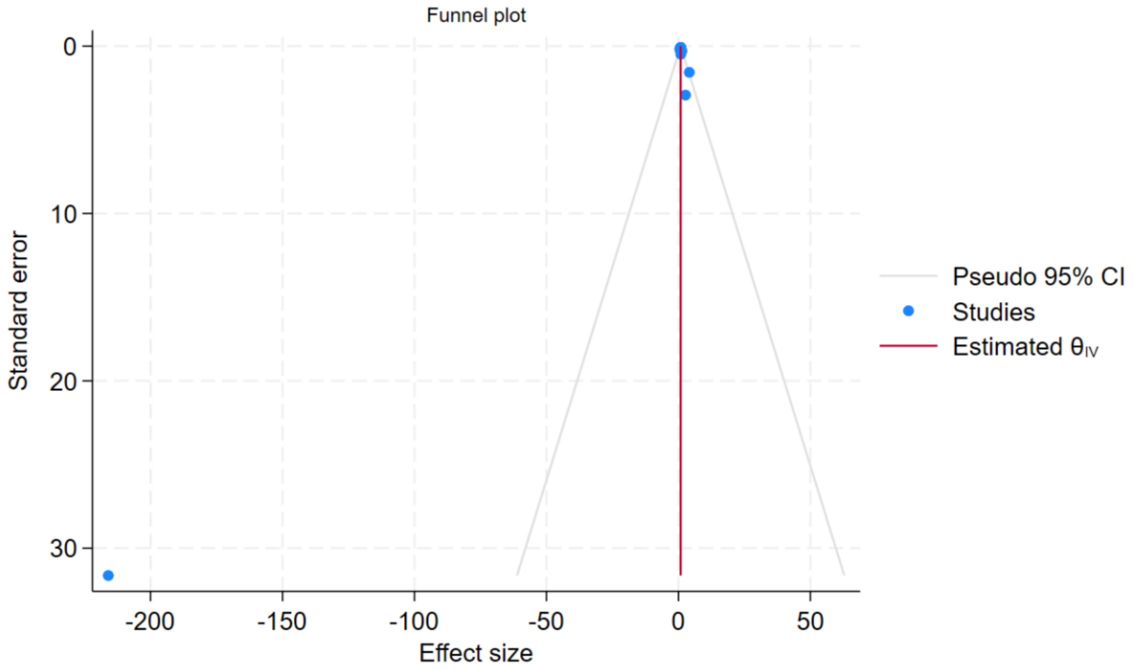

Funnel_macrosomia

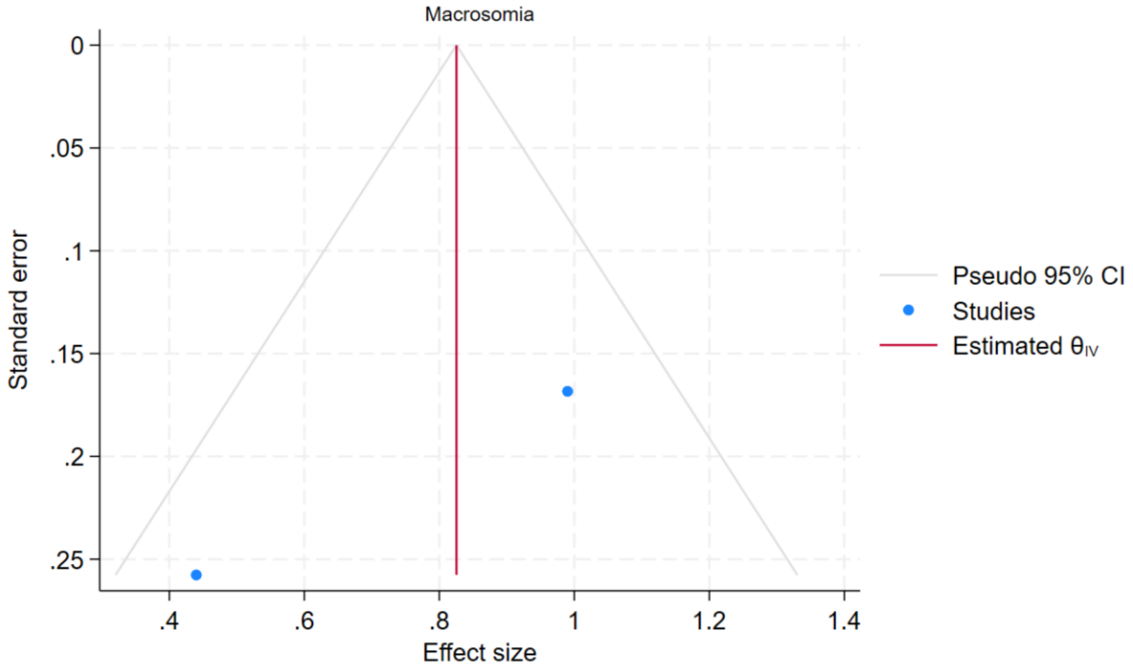

Funnel_neonatal morbidity

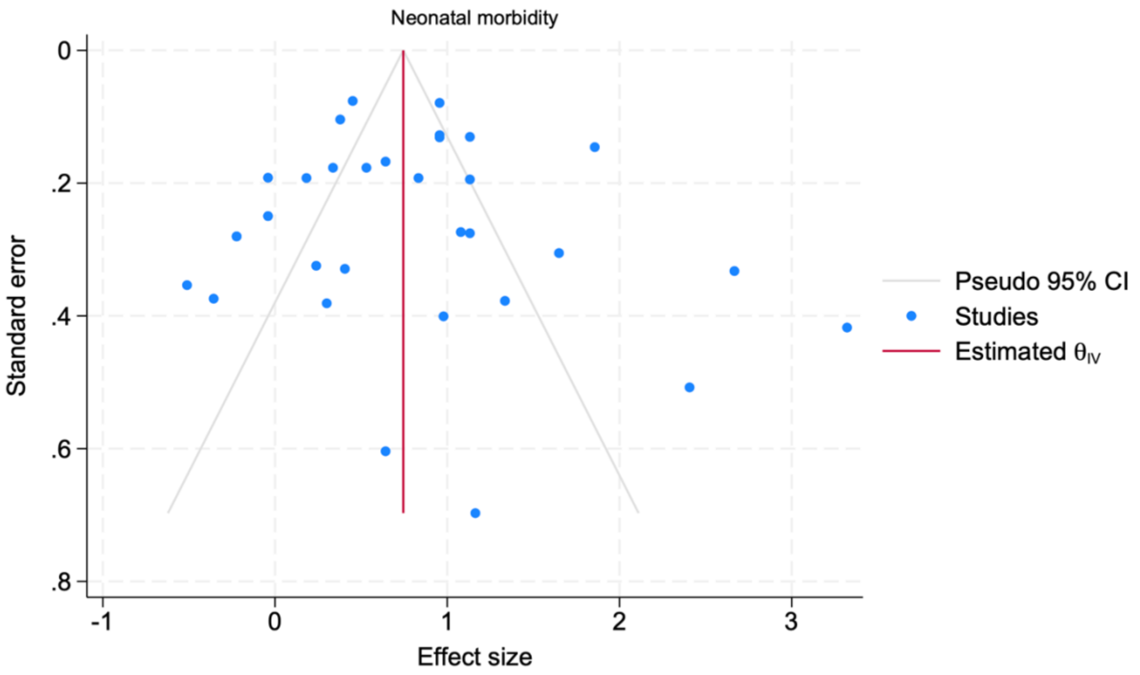

Funnel_neonatal mortality

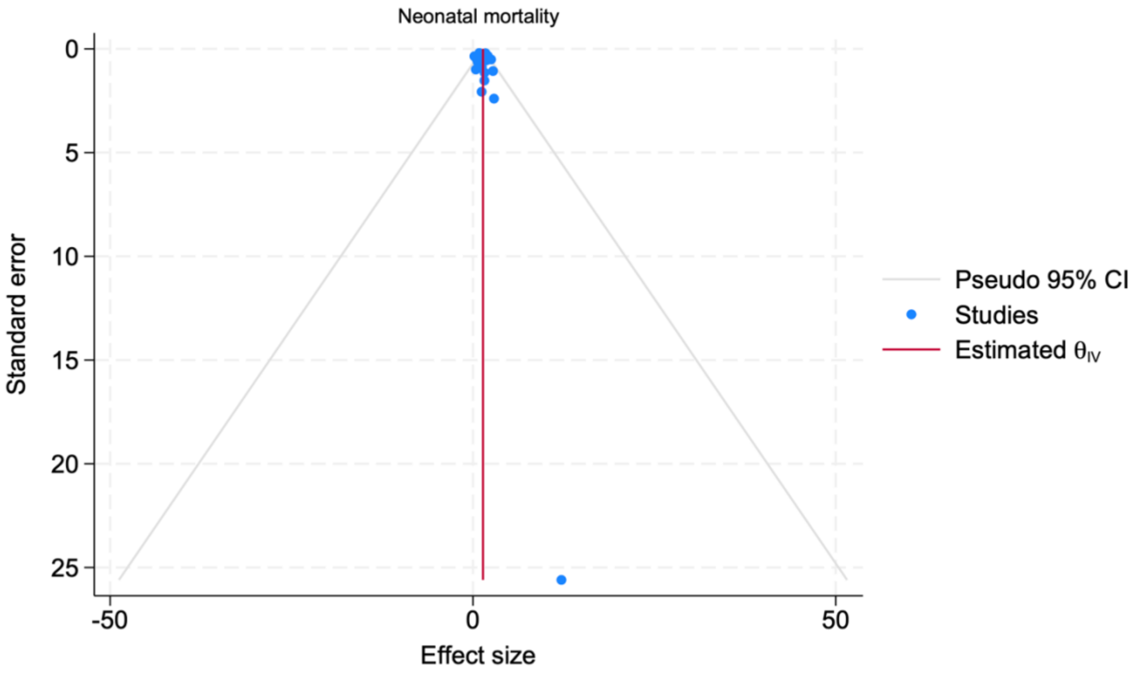

Maternal and perinatal outcomes of Somali migrant women in comparison to host populations in the Global North: a systematic review and meta-analysis Said et al. 2025

Funnel_post term

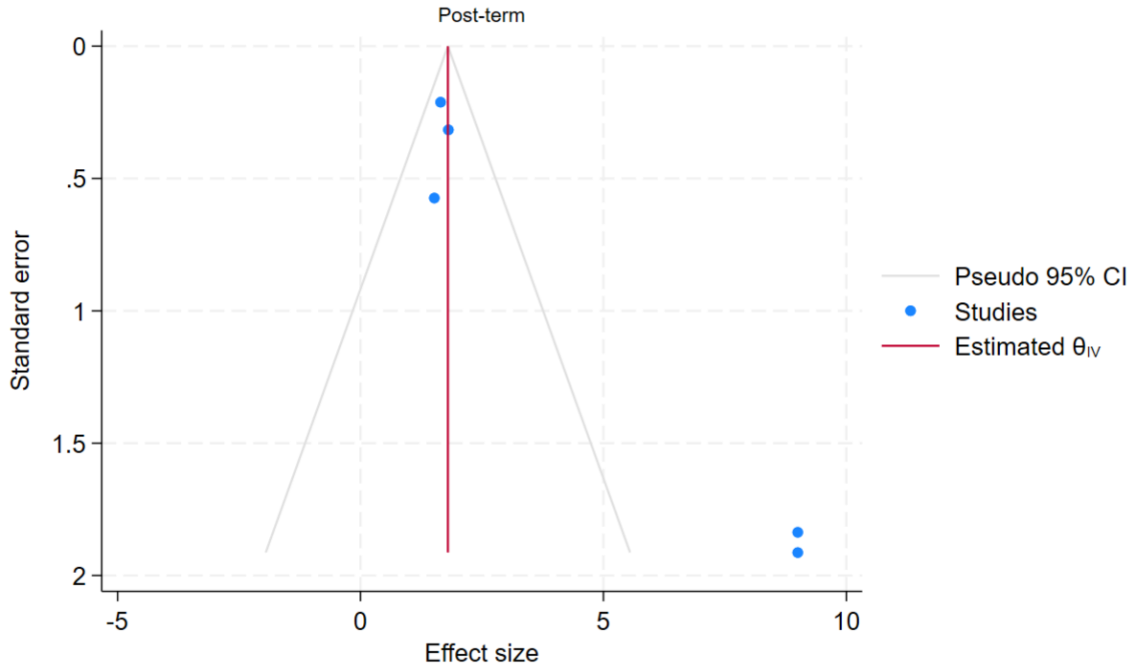

Funnel_Preterm

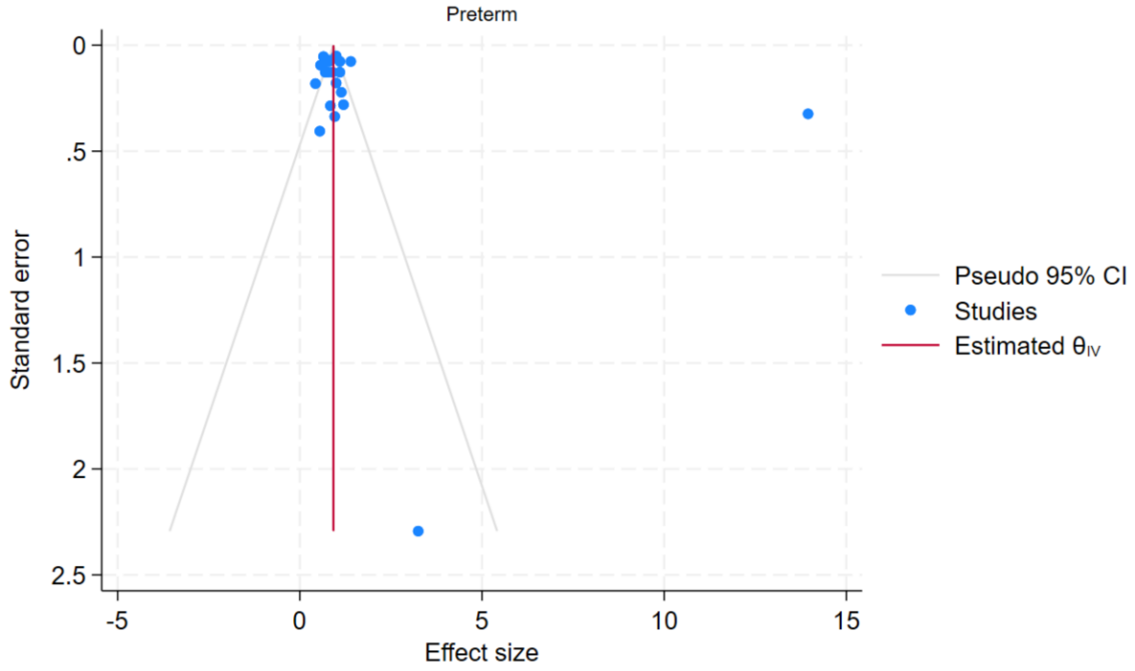

Maternal and perinatal outcomes of Somali migrant women in comparison to host populations in the Global North: a systematic review and meta-analysis Said et al. 2025

Funnel_SGA

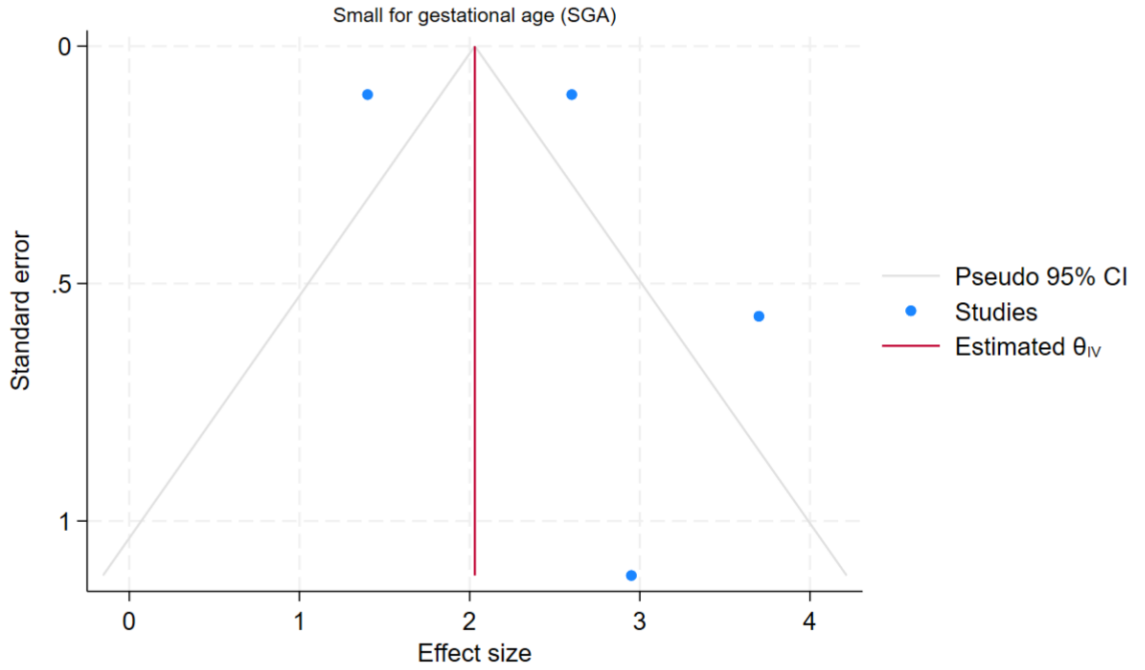

Funnel_SVN

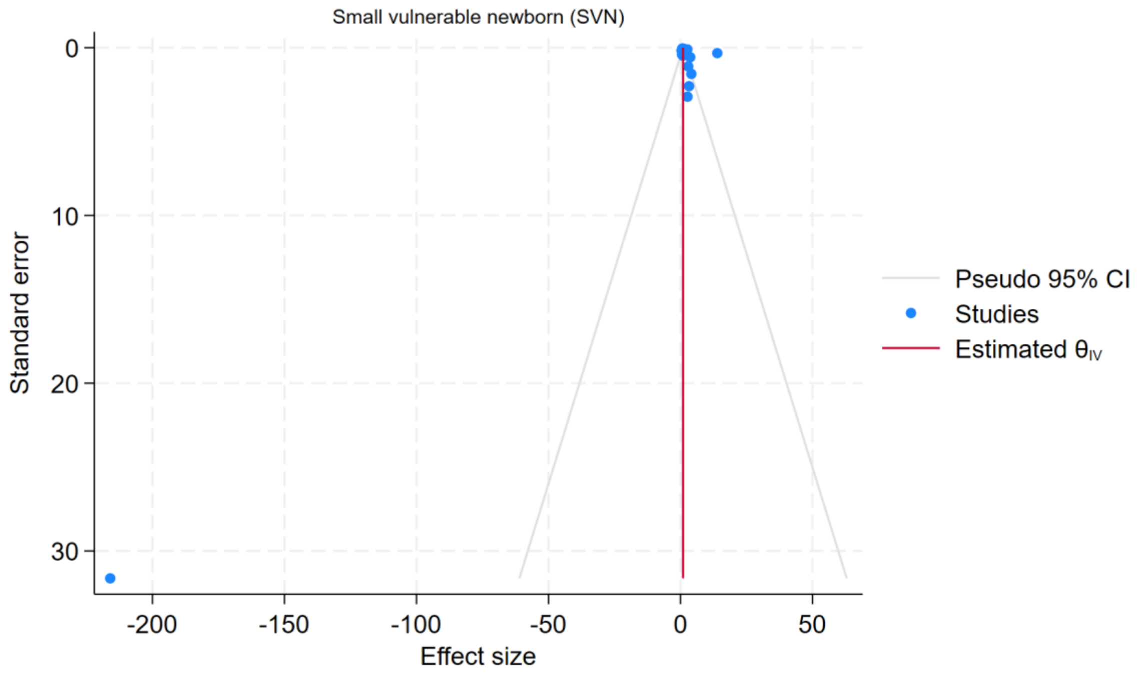

Supplement: Supplementary file 5 — Supplementary Material 5 [file 40748_2025_210_MOESM5_ESM.pdf]
